# Developing erythromycin resistance gene by heavy metals, Pb, Zn, and Co, in aquatic ecosystems

**DOI:** 10.1038/s41598-022-25272-5

**Published:** 2022-12-02

**Authors:** Majid Komijani, Farnaz Eghbalpour, Ebrahim Lari, Nima Shaykh-Baygloo

**Affiliations:** 1 Department of Biology, Faculty of Science, Arak University, Arak, 38156-8-8349 Iran; 2 Department of Molecular Medicine, Golestan University of Medical Sciences, Gorgan, Iran; 3 Department of Biological Sciences, University of Alberta, Edmonton, Alberta T6G 2E9 Canada; 4 Department of Biology, Faculty of Science, Urmia University, Urmia, 5756151818 Iran

**Keywords:** Microbiology, Environmental sciences

## Abstract

Industrial development is the main cause of environmental pollution with various substances such as antibiotics and heavy metals. Many heavy metals with antimicrobial properties could contribute to antibiotic resistance and the emergence of antibiotic resistance genes due to the co-selection phenomenon. The aim of this study was to investigate the concurrent presence and correlation between several heavy metals and the erythromycin resistance genes in six aquatic ecosystems of Iran. Distribution and assessment of 11 erythromycin resistance genes were investigated using specific primers and online enrichment and triple-quadrupole LC–MS/MS. The concentration of heavy metals was measured using inductively coupled plasma atomic emission spectroscopy by Thermo electron corporation. Principal component analysis was performed to globally compare and to determine the similarities and differences among different aquatic ecosystems in different parts of the world in terms of the concentration of zinc and lead in their water. The results of the simple logistic regression analysis for the correlation between erythromycin resistance genes and heavy metals concentrations revealed the most significant correlation between erythromycin resistance genes and Pb concentration, followed by Co and Zn concentrations.

## Introduction

Industrial development is the main cause of environmental pollution with various substances such as antibiotics and heavy metals^1^. As reported by the World Health Organization, the emergence and spread of antibiotic-resistant bacteria (ARB) is considered a serious threat to human health, challenging modern medicine and causing 25,000 and 700,000 deaths annually in the European Union and worldwide, respectively. It is anticipated that antimicrobial resistance will become the main cause of death by 2050 (more than cancer), resulting in enormous economic losses^2–4^. The increasing prevalence of multi-drug resistant pathogens has intensified concerns about antibiotic resistance. The WHO considers antimicrobial resistance as a global public health threat that must be urgently managed, and predicts antibiotic treatment failures will be widespread in the early future^5,6^. Mass production and overuse of antibiotics play an important role in the emergence of ARB^1,7^. Therefore, extensive efforts have been made to limit the use of antibiotics worldwide. However, the limited use of antibiotics has not led to the successful control of the widespread prevalence of antibiotic resistance. Related studies have highlighted the important role of other antimicrobial agents such as heavy metals in the development of antibiotic resistance genes (ARGs)^5,6^.

Heavy metals enter aquatic environments in various ways that are the result of human activities, such as agriculture, livestock and poultry farming, fuel combustion, corrosion of underground pipes, industrial effluents, municipal wastewater, mining, and vehicles^2,7–10^. Heavy metals are a significant threat to the environment and human health due to their high toxicity, durability, and non-degradability^11^. Moreover, many heavy metals with antimicrobial properties (e.g. cadmium, copper, zinc, lead, nickel, mercury, and cobalt) could contribute to antibiotic resistance and the emergence of ARGs due to the co-selection phenomenon^2,3,9,10,12^. ARB containing ARGs caused by the presence of heavy metals proliferate in aquatic environments and can eventually find their way into the human population, livestock, and crops. The horizontal transfer of ARGs to human and animal pathogenic microbes can cause the emergence of new strains of pathogenic ARB. Therefore, it is very important to study the correlation between heavy metals and antibiotic resistance. In addition, bioavailable and highly toxic forms of heavy metals are released into the water and cause a higher level of water pollution. Therefore, the monitoring of heavy metals in aquatic systems is necessary to assess their ecological risks^11^.

In addition to medical use, erythromycin is widely used in the livestock and poultry industry, and aquaculture. Considering the high use of this antibiotic in Iran and the emergence of widespread resistance against it, in this study, the concurrent presence and correlation between several heavy metals and the erythromycin resistance genes (ERGs) were investigated in different aquatic ecosystems of Iran. The study sites were selected based on the potential for high or low human activities impact. Three studied ecosystems were adjacent to cities or industrial centers, including Lake Urmia, Meyghan wetland, and Morreh wetland, and the other three were away from intense human activities, namely Zarivar, Siah Gav, and Keeyow lakes.

## Methods

### Sampling and analysis

**Figure 1:**
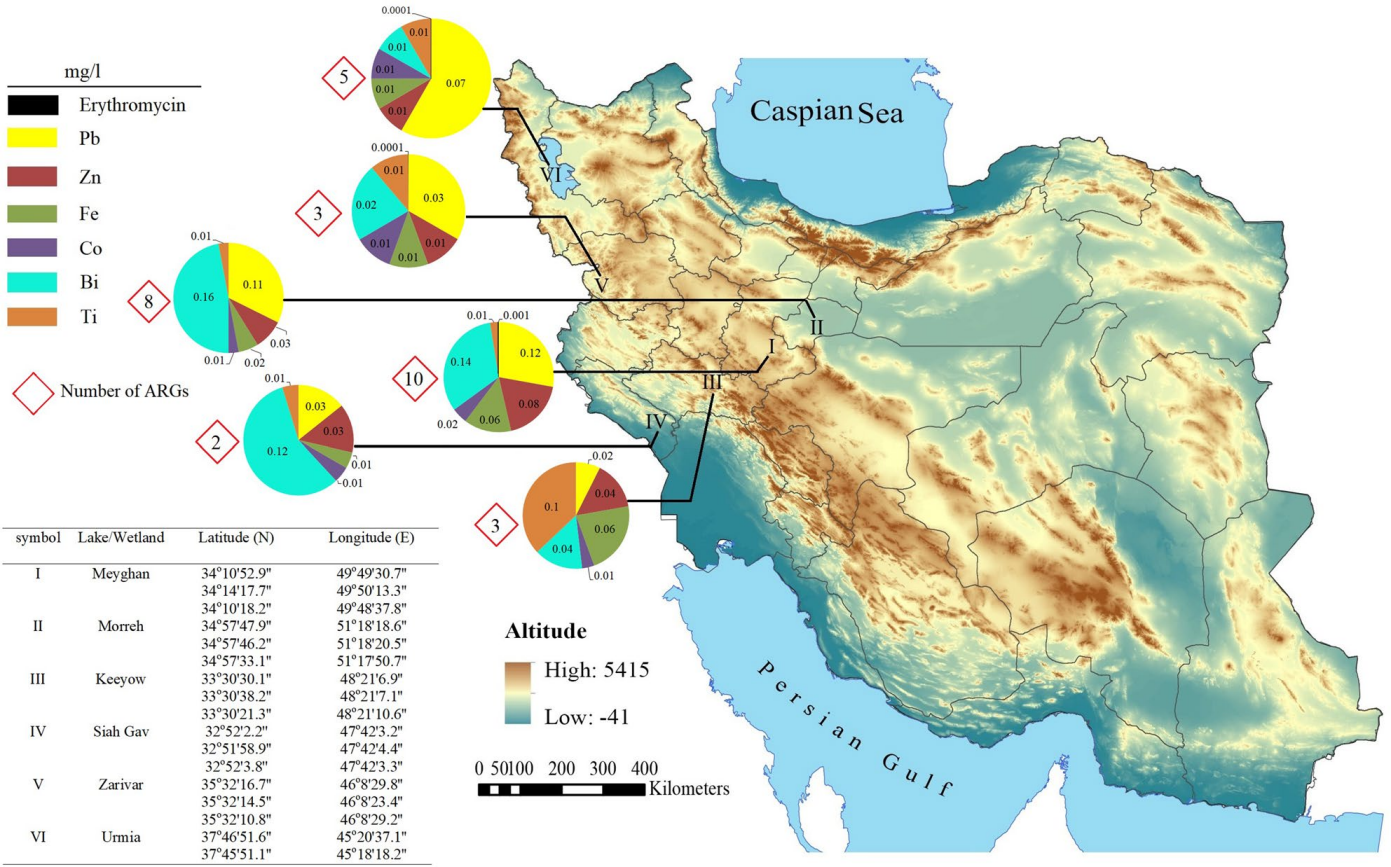
Location, altitude, number of resistance genes, and heavy metals and erythromycin concentration of studied aquatic ecosystems. ArcGIS Desktop 10.8.2 was used to draw the map (https://support.esri.com/en/products/desktop/arcgis-desktop/arcmap/10-8-2).

**Figure 2:**
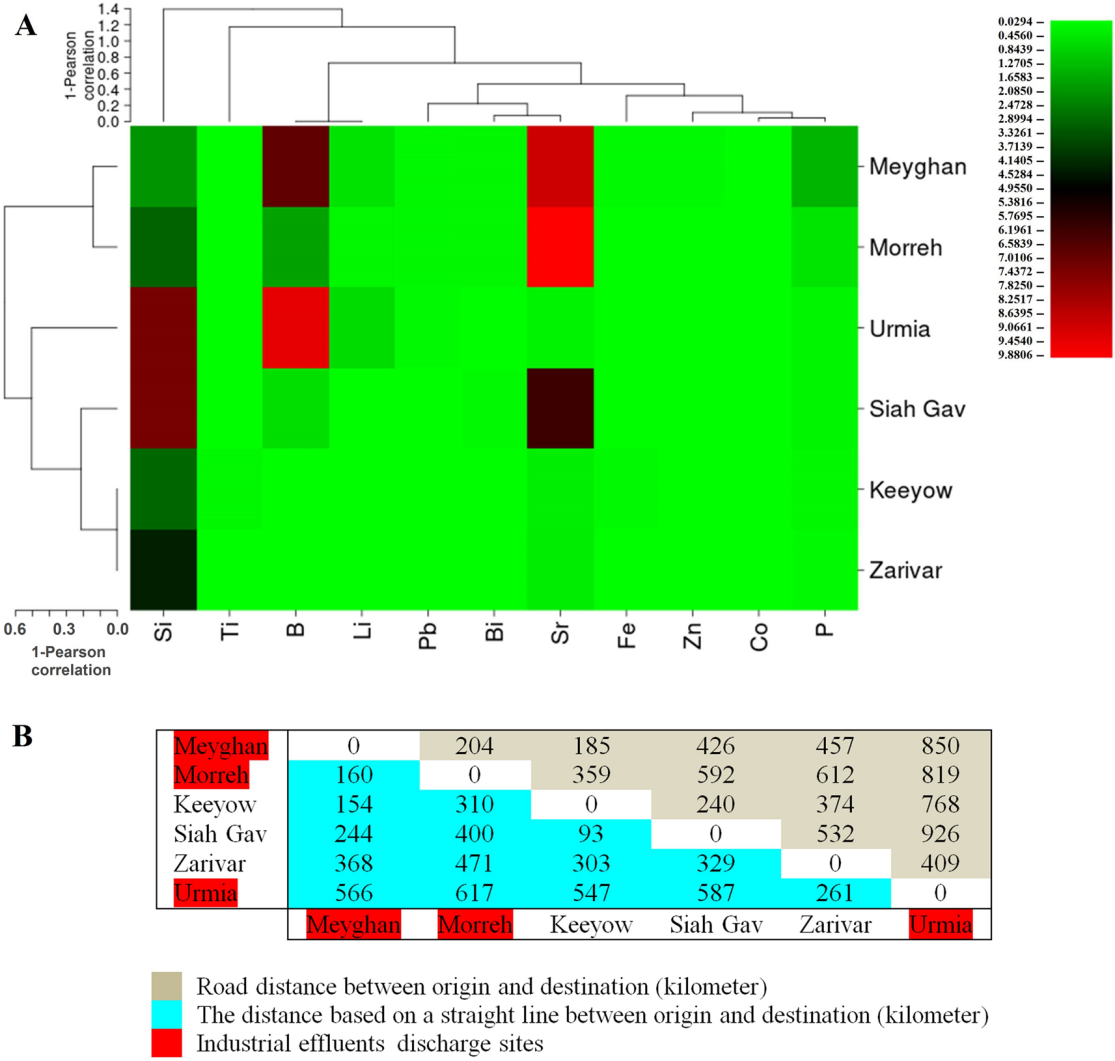
(**A**) The degree of similarity of the studied aquatic ecosystems based on the measured concentrations of elements. (**B**) The distance between aquatic ecosystems.

In the present study, six water bodies were sampled, including Morreh wetland, Lake Urmia, Meyghan wetland, Siah Gav Twin Lakes, Keeyow Lake, and Zarivar Lake. The location and distance between the aquatic ecosystems are shown in Figs. 1 and 2. Three of the study sites were near high population density and industrial areas including Lake Urmia, Meyghan wetland, and Morreh wetland, and the other three were located in areas less impacted by human activities.

Sampling was done from three locations in each study site from a depth of 50–100 cm from mid to late December 2018. The samples were collected in 1 L sterile bottles and transported to the laboratory in less than 3 h. All samples were stored at 4 °C for a maximum of 48 h before use. The samples were centrifuged for 45 s at 5000 g to eliminate the large particles, and the supernatant passed through a 0.2 um pore membrane (JinTeng, China). For DNA isolation, a pre-existing protocol was followed^13^.

### ARGs detection

In order to investigate 11 ARGs that corresponded to erythromycin, they were amplified using specific primers (Table 1). PCR analysis was done in a 25 μl reaction mixture consisting of 2 units *Taq* DNA polymerase, 0.4 μM each primer, 2.5 μl 10 × *Taq* buffer, 2 mM MgCl2, 0.2 mM each dNTPs, and 5 μl DNA template.

**Table 1:** Oligonucleotide primers used to amplify erythromycin resistance genes.

Gene	Sequence	Amplicon size (bp)	References
*erm*B	F: GAAAAGGTACTCAACCAAATA	639	^41^
	R: AGTAACGGTACTTAAATTGTTTA		
*erm*A	F: ATCGGATCAGGAAAAGGACA	537	^42^
	R: AGCCTGTCGGAATTGGTTTT		
*erm*C	F: TGAAATCGGCTCAGGAAAAG	564	^42^
	R: TCGTCAATTCCTGCATGTTT		
*erm*F	F: CGACACAGCTTTGGTTGAAC	309	^43^
	R: GGACCTACCTCATAGACAAG		
*erm*D	F: ATTTTTCCGGACAGCATTTG	520	^42^
	R: ATTCTGACCATTGCCGAGTC		
*erm*T	F: AACCGCCATTGAAATAGACC	480	^42^
	R: GCTTGATAAAATTGGTTTTTGGA		
*erm*X	F: TCCATCATCGACCTTGTGAA	620	^42^
	R: CGCAACCATGATTGTGTTTC		
*mef*A	F: CTGTATGGAGCTACCTGTCTGG	294	^44^
	R: CCCAGCTTAGGTATACGTAC		
*ere*D	F: TTTCCGAAATTGACCTGACC	713	^42^
	R: CACCTTGGCATTTGAGTTTGGT		
*msr*A	F:TCCAATCATTGCACAAAATC	163	^45^
	R:AATTCCCTCTATTTGGTGGT		
*mph*A	F: GTGAGGAGGAGCTTCGCGAG	403	^46^
	R: TGCCGCAGGACTCGGAGGTC		

### Chemical analysis

In water samples, the concentration of B, Bi, Co, Fe, Li, P, Pb, Si, Sr, Ti, and Zn was measured using inductively coupled plasma atomic emission spectroscopy (ICP-AES) by Thermo electron corporation make, (IRIS Intrepid 11 XDL model)^14^.

### Antibiotics assessment

Erythromycin was assessed using online enrichment and triple-quadrupole LC–MS/MS. The advantage of this method over other methods such as X-Ray Fluorescence (XRF) is to reduce the sample volume and simplify the method^15,16^.

### Statistical analyses

Principal component analysis (PCA) was performed to globally compare and to determine the similarities and differences among different aquatic ecosystems in different parts of the world in terms of the concentration of two heavy metals, zinc and lead, in their water^17,18^. For this purpose, all related articles that have examined the concentration of these two metals in the water of different ecosystems around the world were reviewed (Supplementary Table S1)^19–35^. Keywords such as heavy metal, lead, zinc, concentration, pollution, contamination, aquatic ecosystems, water, lake, wetland, river, etc. were used for searching.

Association and correlation tests were done by Microsoft Excel (Microsoft Office Professional Plus, 2016) and GraphPad Prism 8.0.0 (GraphPad Software, San Diego, California USA).

## Results

By examining the presence of 11 ERGs (*ermA*, *ermB*, *ermC*, *ermD*, *ermF*, *ermT*, *ermX*, *ereD*, *mefA*, *mphA*, and *msrA*) in the six aquatic ecosystems, the highest number of resistance genes was observed in Meyghan and Morreh wetlands, containing 10 and 8 ERGs, respectively (Fig. 1). The lowest number (n = 2) of resistance genes was observed in Siah Gav Lake. Among the investigated resistance genes, *ermA* and *ermB* genes were detected in five out of six ecosystems, whereas the *ermX* gene was found only in the Meyghan wetland. Figure 2 shows the results of the ICP analysis, including the concentrations of different elements (B, Bi, Co, Fe, Li, P, Pb, Si, Sr, Ti, and Zn) and the similarity of aquatic ecosystems regarding the concentrations of these elements. Zarivar and Keeyow lakes, which are less affected by industrial activities and municipal wastewater, are very similar in terms of the concentration of the examined elements and are close to each other in Fig. 2. Meyghan and Morreh wetlands, adjacent to industrial cities and affected by industrial and human activities, are also very similar. The highest concentration of Pb was found in Meyghan Wetland, followed by Morreh Wetland, Urmia Lake, Zarivar Lake, and Keeyow Lake = Siah Gav Lake, respectively (Figs. 1 and 2). The concentration of Zn was highest in Meyghan, Keeyow, Morreh = Siah Gav, and Urmia = Zarivar, respectively. Meyghan = Keeyow, Morreh, and Urmia = Siah Gav = Zarivar contained the highest concentrations of Fe, respectively. Bi concentration was equally high in Morreh and Siah Gav, followed by Meyghan, Keeyow, Zavareh, and Urmia, respectively. Co and Ti were the maximum levels in the Meyghan and Keeyow ecosystems, respectively, and similar concentrations of these two heavy metals were recorded in the other ecosystems.

The results of the simple logistic regression analysis for the correlation between ERGs and heavy metals concentrations revealed the most significant correlation between ERGs and Pb concentration (*P*-value = 0.0001), followed by Co (*P*-value = 0.0103) and Zn (*P*-value = 0.0092) concentrations. As shown in Fig. 1, the increasing concentrations of the Pb, Co, and Zn increased the diversity of ERGs in the studied ecosystems. The results showed no significant correlation between Fe, Bi, and Ti concentrations with the ERGs.

**Figure 3:**
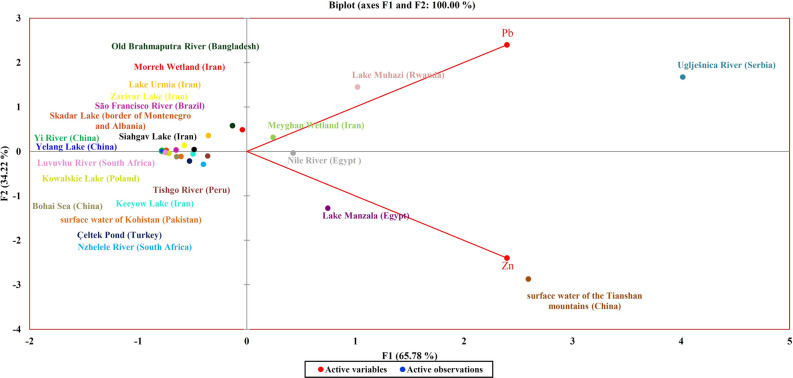
Comparison of different aquatic ecosystems (aquatic ecosystems studied herein and those from other studies worldwide) in terms of Pb and Zn concentrations in their water using PCA.

The concentrations of Pb and Zn have been measured in numerous other aquatic ecosystems worldwide. Reports have shown that the most resistance genes are related to these two heavy metals. In this study, therefore, a global comparison of the water of different aquatic ecosystems (aquatic ecosystems investigated in the present study and those of other studies) was performed based on the concentrations of Pb and Zn using PCA (Fig. 3). Although there was a significant correlation between Co concentration and erythromycin resistance gene, this element has been analyzed in few studies, and it was not possible to compare it in different aquatic ecosystems, it was not used for PCA plotting. PCA resulted in two corresponding factor F1 and F2, with 100% cumulative variance. Corresponded variance of F1 and F2 were 65.78% and 34.22% respectively. The water of the Uglješnica River (in Serbia), which is located on the top right of Fig. 3, contains the highest concentration of Pb among the compared aquatic ecosystems, and the water of the Tianshan Mountains (China), which is located on the bottom right of Fig. 3, contains the highest concentration of Zn. Lake Yelang (China) and the Yi River (China), with the lowest concentrations of Pb and Zn, are located on the horizontal midline in the leftmost part of the figure. (Ecosystems containing a higher Pb concentration than Zn are located at the top of the midline and shift further to the right with higher concentrations of Pb. Contrastingly, ecosystems in which the Zn concentration is higher than Pb are located at the bottom of the midline and shift further to the right with higher concentrations of Zn. Also, ecosystems with equal concentrations of these two heavy metals in their water are located on the horizontal midline).

## Discussion

A major mechanism to acquire antibiotic resistance under the selective pressure of heavy metals is co-resistance. Co-resistance occurs when two or more resistance genes of different antimicrobial agents are placed on a mobile genetic element, such as a plasmid or transposon, inducing multiple resistance^3,36^. The physical connection of an ARG and a heavy metal resistance gene (HMRG) on the same genetic element causes bacterial resistance to both antimicrobial agents when the genetic element is acquired. Even when only one of the co-selecting factors (i.e. antibiotic or heavy metal) is present^3,37^. Therefore, the presence of heavy metals in the environment can induce antibiotic resistance in the absence of antibiotics in the bacterial population. Plasmids with co-selection ability resulting in the concurrence of ARG and HMRG in bacteria have been reported in several studies^3,10,12^. Another mechanism that causes co-selection is cross-resistance, which occurs when a single mechanism (e.g. an efflux pump) simultaneously induces resistance to several different antimicrobial agents such as antibiotics and heavy metals^3,12^. In addition to co-resistance and cross-resistance, co-regulatory mechanisms can also cause co-selection. This type of resistance occurs when several resistance genes, which cause resistance to various antimicrobial agents, are controlled by a single regulatory gene^3,12,38^. The correlation between heavy metal concentrations and antibiotic resistance has been shown in several studies^3,9,39^. Meaning that an increase in the concentration of some heavy metals (most of which are toxic to microorganisms at high concentrations) in the environment result in the survival of only microorganisms that possess resistance genes for these metals or are able to obtain resistance genes from the environment. The proliferation of resistant microorganisms leads to the increase and spread of HMRGs in the environment. The increase in HMRGs will amplify and expand ARGs as a result of the co-selection phenomenon. Some studies have reported correlation between the emergence of multi-drug resistant pathogens and resistance to several heavy metals^3^. An increase in ARGs in an environment containing high concentrations of heavy metals may also result from the proliferation of microorganisms that previously acquired ARGs (due to overuse of antibiotics and their entry into the environment); they contain HMRGs owing to the co-selection phenomenon and are able to survive and proliferate in environments containing high concentrations of heavy metals^9^. In the present study, the most ERGs (10 of the 11 studied resistance genes) were observed in the Meyghan wetland, followed by the Morreh wetland (8 ERGs) and Lake Urmia (5 ERGs). The least numbers of ERGs were detected in Siah Gav, Zarivar, and Keeyow lakes. Consistent with a similar study^40^, the present results indicate high levels of heavy metals in the Meyghan wetland, Morreh wetland, and Lake Urmia compared to Siah Gav, Zarivar, and Keeyow lakes. Various industrial effluents and treated municipal wastewater (though it may not be complete) enter Meyghan wetland, Morreh wetland, and Lake Urmia, increasing the concentrations of heavy metals in these three aquatic ecosystems. Since the elevated concentrations of heavy metals can increase ARGs through co-selection^3,9,39^, it seems that the high numbers of ERGs are related to the high levels of heavy metals in these three aquatic ecosystems. However, ARGs might have entered these three aquatic ecosystems along with human and animal pathogens present in effluents from human health centers, agricultural industries, and livestock, poultry, and fish culture industries, which use different antibiotics to treat and prevent bacterial infections. Given that these three water bodies are classified as saline aquatic ecosystems, and Lake Urmia is one of the most saline lakes in the world, the replication of human and animal pathogens and thus the amplification of ARGs are not possible in these ecosystems. Therefore, the high numbers of ARGs in these three aquatic ecosystems compared to the other three aquatic ecosystems most probably result from the high concentrations of heavy metals and the co-selection event.

In various studies, the most number of resistance genes in the ecosystems have been attributed to Pb, Zn, and Co heavy metals^3^, suggesting that these metals play an important role in the development of antibiotic resistance through co-selection. In the present study, Pb, Zn, and Co were significantly correlated with ERGs. The observed increase in the abundance of antibiotic resistance was most likely resulted from the high heavy metal concentrations and the co-selection phenomenon in the investigated aquatic ecosystems. Erythromycin is widely used to treat infections of humans, and livestock, poultry, and aquatic animals. Infections caused by bacteria resistant to erythromycin can seriously complicate the treatment process and cause increased mortality. The CDC (Centers for Disease Control and Prevention) report (2019) indicates that increasing resistance to erythromycin, especially in people who are allergic to penicillin, complicates the treatment of streptococcal infections and increases mortality from infections^47^.

As mentioned above, the increasing concentration of heavy metals causes the emergence and spread of resistance genes against them, and as a result of the co-selection phenomenon, causes the emergence and spread of ARG in aquatic ecosystems^2,3,9,10,12^. Accordingly, by comparing aquatic ecosystems in which the concentration of heavy metals and the presence of ARGs have been investigated, with those in which only the concentration of heavy metals has been investigated, the presence and spread of ARGs can be predicted in ecosystems with known heavy metal concentrations. In this investigation, the water of the studied ecosystems was compared with that of several other aquatic ecosystems in terms of Pb and Zn concentrations. Since Pb and Zn influence the spread of HMRGs genes (and consequently the spread of ARGs), and the concentrations of these two heavy metals were significantly correlated with the presence of ERGs in aquatic ecosystems, the presence of ARGs can be predicted in the other compared ecosystems.

## Conclusion

Due to the correlation between HMRGs and ARGs, the increased concentrations of heavy metals in aquatic ecosystems due to human activity and inattention to their entry into the environment lead to the spread of ARGs, resulting in the emergence of multi-drug resistant pathogens, which will, in turn, have irreversible consequences. Therefore, the excessive use of antibiotics and the increase of other antimicrobial agents (e.g. heavy metals) in the environment should be under surveillance to prevent the occurrence of multi-drug resistant bacteria and successfully cure bacterial infections using antibiotics.

## Supplementary Information

Supplementary Information.

## Data Availability

All data generated or analysed during this study are included in this published article (and its Supplementary Information files).
