# Supplementary material for: Developing erythromycin resistance gene by heavy metals, Pb, Zn, and Co, in aquatic ecosystems
Source: Sci Rep. 2022 Dec 2;12:20797. doi: 10.1038/s41598-022-25272-5 (PMC9718742; doi:10.1038/s41598-022-25272-5)
Supplement: Supplementary file 1 — Supplementary Information. [file 41598_2022_25272_MOESM1_ESM.pdf]

Place-name	Country	Concentration of Pb and Zn (mg/L)	Reference
Skadar Lake	border of Montenegro and Albania	<0.005, 0.004	[24]
Nile River (winter)	Egypt (Cairo)	0.105, 0.139	[23]
Tishgo River	Peru	0.028, 0.060	[21]
Lake Manzala	Egypt	0.022, 0.311	[19]
Old Brahmaputra River	Bangladesh	0.11, 0.01	[20]
Uglješnica River	Serbia	0.582, 0.346	[27]
surface water of the Tianshan Mountains	China	0.045, 0.689	[35]
Yi River	China	0.0004, 0.0009	[25]
Çeltek Pond	Turkey	0.0027, 0.054	[29]
Kowalskie Lake reservoir	Poland	0.0012, 0.014	[31]
Groundwater of Kohistan region	Pakistan	0.0096, 0.951	[28]
surface water of Kohistan region	Pakistan	0.005, 0.034	[28]
Yelang Lake	China	0.0003, 0.0006	[34]
Lake Muhazi	Rwanda	0.292, 0.041	[30]
Luvuvhu River	South Africa	0.0001, 0.0082	[26]
Bohai Region	China	0.001, 0.03	[33]
Nzhelele River	South Africa	0.0078, 0.076	[22]
lower São Francisco River basin	Brazil	0.014, 0.013	[32]

Supplementary Table S1. Concentration of two heavy metals, Pb and Zn, in the water of various aquatic ecosystems around the world.
